# Supplementary material for: Detection of asymptomatic Leishmania infection in blood donors at two blood banks in Ethiopia
Source: PLoS Negl Trop Dis. 2023 Mar 9;17(3):e0011142. doi: 10.1371/journal.pntd.0011142 (PMC10030029; doi:10.1371/journal.pntd.0011142)
Supplement: S1 Table — DAT: direct agglutination test; ELISA: PCR: polymerase chain reaction; RDT: rapid diagnostic test (DOCX) [file pntd.0011142.s001.docx]

**S1 Table. Agreement and Kappa score for the different Leishmania tests**

| **Test combination** | **Agreement (%)** | **Kappa score** |
| --- | --- | --- |
| rK39 ELISA & rK39 RDT | 92.3 | 0.269 |
| rK39 ELISA & DAT | 94.2 | -0.009 |
| rK39 ELISA & PCR | 92.2 | 0.045 |
| rK39 RDT & PCR | 94.9 | 0.049 |
| rK39 RDT & DAT | 97.0 | 0.035 |
| DAT & PCR | 97.0 | 0.035 |

DAT: direct agglutination test; ELISA: PCR: polymerase chain reaction; RDT: rapid diagnostic test
