# Supplementary material for: Detection of asymptomatic Leishmania infection in blood donors at two blood banks in Ethiopia
Source: PLoS Negl Trop Dis. 2023 Mar 9;17(3):e0011142. doi: 10.1371/journal.pntd.0011142 (PMC10030029; doi:10.1371/journal.pntd.0011142)
Supplement: Attachment — Submitted filename: Blood_Tx_Reply_v2.docx [file pntd.0011142.s002.docx]

Dear Dr. van Griensven,

Thank you very much for submitting your manuscript "Detection of asymptomatic Leishmania infection in blood donors at two blood banks in Ethiopia: should blood donors be systematically screened?" for consideration at PLOS Neglected Tropical Diseases. As with all papers reviewed by the journal, your manuscript was reviewed by members of the editorial board and by several independent reviewers. In light of the reviews (below this email), we would like to invite the resubmission of a significantly-revised version that takes into account the reviewers' comments.

This paper is interesting because data on persons infected asymptomatically ba Leishmania parasites are very scarce. But the authors should ask advice for statistical analysis of the data as recommended by reviewer 2. Would transfer to PLoS One be an option for the authors?

**Dear Editor,**

**We have asked advice of a statistician. We very much appreciate the comments of the reviewers, as they helped to substantially improve the paper. Moreover, we think we generally managed to address their concerns. We also think this paper would fit very well in a journal dedicated to neglected tropical diseases, as if focusses on one of the (fatal) neglected tropical diseases. Given the recent ambition to go for elimination of visceral leishmaniasis (VL) in East Africa, our findings are relevant and timely, as a good understanding of all modes of transmission of VL is vital to achieve this. As such, we think our paper would be of much interest to the readers of your journal.**

**Finally, we think the quality of the research is high and merits publication in a journal like PLOS NTDs. This study was conducted within a research set-up geared towards GC(L)P compliant clinical research in Ethiopia, including clinical trials. Over the last ten years, substantial investments have been done in internal and external quality control of the laboratory involved in this study. As to the conduct of the study, the site has been supported by the ITM clinical trials unit for over a decade, to engender best research practices.**

We cannot make any decision about publication until we have seen the revised manuscript and your response to the reviewers' comments. Your revised manuscript is also likely to be sent to reviewers for further evaluation.

When you are ready to resubmit, please upload the following:

[1] A letter containing a detailed list of your responses to the review comments and a description of the changes you have made in the manuscript. Please note while forming your response, if your article is accepted, you may have the opportunity to make the peer review history publicly available. The record will include editor decision letters (with reviews) and your responses to reviewer comments. If eligible, we will contact you to opt in or out.

[2] Two versions of the revised manuscript: one with either highlights or tracked changes denoting where the text has been changed; the other a clean version (uploaded as the manuscript file).

Important additional instructions are given below your reviewer comments.

Please prepare and submit your revised manuscript within 60 days. If you anticipate any delay, please let us know the expected resubmission date by replying to this email. Please note that revised manuscripts received after the 60-day due date may require evaluation and peer review similar to newly submitted manuscripts.

Thank you again for your submission. We hope that our editorial process has been constructive so far, and we welcome your feedback at any time. Please don't hesitate to contact us if you have any questions or comments.

Sincerely,

Gabriele Schönian

Academic Editor

PLOS Neglected Tropical Diseases

Charles Jaffe

Section Editor

PLOS Neglected Tropical Diseases

***********************

This paper is interesting because data on persons infected asymptomatically ba Leishmania parasites are very scarce. But the authors should ask advice for statistical analysis of the data as recommended by reviewer 2. Would transfer to PLoS One be an option for the authors?

Reviewer's Responses to Questions

<b>Key Review Criteria Required for Acceptance?</b></br></br> As you describe the new analyses required for acceptance, please consider the following:</br></br>

<b>Methods</b></br></br>

-Are the objectives of the study clearly articulated with a clear testable hypothesis stated?</br> -Is the study design appropriate to address the stated objectives?</br> -Is the population clearly described and appropriate for the hypothesis being tested?</br> -Is the sample size sufficient to ensure adequate power to address the hypothesis being tested?</br> -Were correct statistical analysis used to support conclusions?</br> -Are there concerns about ethical or regulatory requirements being met?</br></br>

Reviewer #1: Methods are clearly described

Reviewer #2: -Are the objectives of the study clearly articulated with a clear testable hypothesis stated? YES -Is the study design appropriate to address the stated objectives? YES -Is the population clearly described and appropriate for the hypothesis being tested? YES -Is the sample size sufficient to ensure adequate power to address the hypothesis being tested? Comment were provided in the review.

-Were correct statistical analysis used to support conclusions? Comment were provided in the review.

-Are there concerns about ethical or regulatory requirements being met? Comments were provided

Reviewer #3: Sample size calculation

Lines 132-136.

I questioned the authors’ sample size calculation for two study sites, that is, recruiting an equal proportion of study participants given Metemma is one of the classical VL endemic areas in Ethiopia while Gonder city and surrounding areas are not yet recognized as one of VL endemic foci. In addition, the authors used a 15.6% infection rate reported in Brazil to estimate the number of study participants to be enrolled in this cross-sectional study. I strongly believe this is not appropriate given that published data (PMID: 33430946 plus the references 14-16 cited by authors) regarding the rate of asymptomatic VL in Ethiopian VL endemic foci are available. Blood donors usually represent apparently healthy individuals and are an appropriate target to study the rate of asymptomatic infection.

**Reply:**

**As this is the first study on this in East-Africa, we indeed found it important to get reliable data from a well-known VL endemic area. However, with the current expansion of VL in Ethiopia, and with the increasing mobility between VL endemic and non-VL endemic areas, this classical classification as VL endemic or non-endemic might not hold anymore. Moreover, in the area, Gondar is the main site for blood collection. Hence, as a pilot study and the first ever conducted in East-Africa, we decided to aim for reliable data from both types of settings, to inform further work.**

**We indeed could have used data on asymptomatic *Leishmania* infection from Ethiopia to inform the sample size calculation. However, there are several issues with the available data (either differences in study population, sampling strategy or the type of Leishmania tests used)**

1) **PMID: 33430946: due to the specific sampling procedure (not random or consecutive) in this study, no reliable prevalence estimate can be obtained that could be extrapolated to donors**

**“Following LST-reactive individuals as a focal point, dogs were sampled for serological tests, rK39 and DAT. Also, 253 human blood samples, 67 purposively from LST-reactive and 185 randomly from non-reactive individuals, were tested by DAT and rK39.”**

**REF 14-16**

**<https://pubmed.ncbi.nlm.nih.gov/23029576/>**

**This study only included children**

**<https://pubmed.ncbi.nlm.nih.gov/22556076/>**

**This study only included children**

**<https://pubmed.ncbi.nlm.nih.gov/31881021/>**

**The study population/design of this study would probably be the best match to provide prevalence data on asymptomatic *Leishmania* infection to inform the sample size calculation for our study, however this study used LST, next to DAT, to identify asymptomatic infection. LST is rather an indicator of good immunity and hence would rather be an indicator of a lower likelihood to host parasites.**

**Other references**

**<https://pubmed.ncbi.nlm.nih.gov/29774295/>**

**This study used LST, next to rK39 RDT, to identify asymptomatic infection. LST is rather an indicator of good immunity and hence would rather be an indicator of a lower likelihood to host parasites.**

**One study we conducted in Metema used rK39, DAT, PCR and KATEX, and hence would be fairly comparable found the following data:**

**<https://pubmed.ncbi.nlm.nih.gov/31593563/>**

**“The prevalence of asymptomatic *Leishmania* infection at baseline was 12.8%**

**in males and 4.2% in females, with the highest prevalence (17.9%) found in males aged**

**between 28 and 38 years old”**

**However, this study only included HIV-infected patients.**

**In conclusion, we did not find any study sufficiently comparable to inform our sample size calculation. Moreover, we also did not know to what extent individuals presenting for blood donation differ (eg in proportion of males/females, age) from studies included in community-based surveys.**

**However, if using the latter, one would extrapolate a prevalence of Leishmania infection of around 10-15% for a study population with a male/female ratio and age distribution as ours, which is fairly similar to the estimate we used for the sample size calculation. While we acknowledge there are also limitations with that, the study from Brazil at least was comparable in study population, in the sense that it included blood donors, and it used a sensitive serological marker.**

**If we would have used the estimates from the studies from Ethiopia, generally a bit lower than the 15.6% we used in the sample size calculation, an increased precision would be obtained with the planned sample size. So in that sense, our sample size is a bit larger than if it would have been based on lower prevalence estimates.**

**We have added the following information: “****Using a prevalence of 10% as could be expected from some studies enrolling healthy individuals in Ethiopia, a precision of 4.1% would be obtained.”**

<b>Results</b></br></br>

-Does the analysis presented match the analysis plan?</br> -Are the results clearly and completely presented?</br> -Are the figures (Tables, Images) of sufficient quality for clarity?</br></br>

Reviewer #1: Results are clearly described

Reviewer #2: -Does the analysis presented match the analysis plan? NO. See comments -Are the results clearly and completely presented? No, see comments -Are the figures (Tables, Images) of sufficient quality for clarity? NO (tables). See comments

Reviewer #3: Prevalence of asymptomatic infection Lines 207-209 The authors of the present study found low seropositivity using DAT in comparison to the rK39 rapid test (RDT) and rK39 ELISA. Contrary to their findings, several studies [PMID: 28446246; PMID: 17942129] revealed the poor sensitivity of rK39-based serologic tests in Ethiopia which is partially related to the high genetic diversity of the L. donovani strains circulating in Ethiopia. On the hand, DAT is reported to be sensitive enough and has been recommended to be performed in those samples that were found to be negative by the rK39 test. Thus, the authors need to discuss the reason why they found relatively higher seropositivity by rK39 ELISA (5%) and rK39 RDT (3%) than by DAT (0.5%)?

**Reply: Thank you for this comment, this is a fair point. The increased detection of asymptomatic infection with rK39 RDT/ELISA vs DAT is indeed consistent within and outside of Ethiopia.**

**The same was observed in another study in HIV patients we conducted in Metema (<https://pubmed.ncbi.nlm.nih.gov/31593563/>) and confirmed in a larger study in another study area**

**(<https://clinicaltrials.gov/ct2/show/NCT03013673>). We observed the same in studies from colleagues at ITM in the Indian subcontinent, particularly for rK39 ELISA vs DAT.**

**(data presented at the 7 the World Leish conference in Colombia, August 2022 - Abstract O39-04: COMBINING HUMAN SEROSURVEILLANCE WITH MOLECULAR XENOMONITORING IN TWO ENDEMIC VILLAGES FOR VISCERAL LEISHMANIASIS SURVEILLANCE IN SARAN, BIHAR, INDIA; manuscript in preparation.**

**Indeed, most data on the performance of DAT and rK39-based tests come from symptomatic VL, and as to East-Africa, the better performance of DAT to diagnose VL does not automatically mean this test would perform better in diagnosing asymptomatic infection. As to the potential reasons behind that, very little is published. As thresholds for DAT positivity have historically been put to diagnose VL and not asymptomatic infection, this could contribute – and we have referred to that now in the discussion – other factors beyond current understanding could contribute as well.**

--------------------

<b>Conclusions</b></br></br>

-Are the conclusions supported by the data presented?</br> -Are the limitations of analysis clearly described?</br> -Do the authors discuss how these data can be helpful to advance our understanding of the topic under study?</br> -Is public health relevance addressed?</br></br>

Reviewer #1: Conclusions are clearly described, and supported by the data

Reviewer #2: -Are the conclusions supported by the data presented? Yes -Are the limitations of analysis clearly described? NO -Do the authors discuss how these data can be helpful to advance our understanding of the topic under study? NO -Is public health relevance addressed? YES

Reviewer #3: Discussion

Lines 266-273

Authors recommend the inclusion of the serologic rK39 RDT test in the systematic screening of blood donors in East African settings emphasizing targeting male donors as a logistically feasible approach. Implementing this would still be unlikely given (i) most blood donors in Ethiopia, if not all East African countries, are males plus (ii) the sensitivity and specificity of the recommended rK39 RDT in Ethiopia, including Metemma, are poor and may not be a reliable diagnostic test to rule out Leishmania infection. Additionally, antibody detection in blood and products from those donors may not necessarily indicate the potential to pose a sensible threat to blood recipients. Excluding positive male donors from donating blood on the basis of serologic tests, therefore, would result in major limitation on blood availability, thus causing more harm than benefit. Given the difficulty in screening-test selection, and testing cost as suggested by authors, looking for cost-effective Leishmania parasite inactivation and/or filtration methods using riboflavin and ultraviolet light and filtration leukodepletion filters, respectively of the blood and blood products derived from blood donors residing in VL endemic areas can be considered as effective and efficient alternatives to provide safe and immediate blood transfusion access to the needing blood recipients.

--------------------

**Reply: Thank you for this very valuable thoughts. We have given this a lot of further thought ourselves and have also discussed with other Ethiopian colleagues not involved in this publication.**

**Basically, we agree with you that it would be premature to recommend systematic screening of blood donors for Leishmania infection already at this stage, as the downstream consequences of that would indeed probably have more harm then benefit. We have rephrased relevant sections in line with that throughout the paper, and have also integrated your suggestion to look into inactivation methods.**

<b>Editorial and Data Presentation Modifications?</b></br><br/> Use this section for editorial suggestions as well as relatively minor modifications of existing data that would enhance clarity. If the only modifications needed are minor and/or editorial, you may wish to recommend “Minor Revision” or “Accept”.

Reviewer #1: (No Response)

Reviewer #2: See comments below

Reviewer #3: Specific comments

Title:

Authors may need to change their manuscript title as their findings could not provide sufficient data for the inclusion of proper screening methods for screening blood donors.

I recommend “Prevalence of Leishmania donovani antibodies and DNA among blood donors at two blood banks in Northwest Ethiopia”.

**We have removed the second part of the title, as we agree we do not have sufficient evidence to recommend systematic screening.**

**We however would prefer to keep the term “asymptomatic Leishmania infection” in the title for the reasons mentioned below:**

**The term “asymptomatic *Leishmania* infection” is consistently used by many studies, despite being aware there are limitations related to this term, eg:**

**<https://pubmed.ncbi.nlm.nih.gov/36040931/>**

**Prevalence and determinants of asymptomatic Leishmania infection in HIV-infected individuals living within visceral leishmaniasis endemic areas of Bihar, India**

**Gadisa E, Custodio E, Canavate C, Sordo L, Abebe Z, Nieto J, et al. Use‑ fulness of the rK39-immunochromatographic test, direct agglutination test, and leishmanin skin test for detecting asymptomatic Leishmania infection in children in a new visceral leishmaniasis focus in Amhara State, Ethiopia. Am J Trop Med Hyg. 2012;86:792–8. https://doi.org/10. 4269/ajtmh.2012.11-0196. 25.**

**https://journals.plos.org/plosntds/article?id=10.1371/journal.pntd.0001813**

**Factors Associated with Leishmania Asymptomatic Infection: Results from a Cross-Sectional Survey in Highland Northern Ethiopia**

**Ayehu A, Aschale Y, Lemma W, Alebel A, Worku L, Jejaw A, et al. Seroprevalence of asymptomatic Leishmania donovani among laborers and associated risk factors in agricultural camps of West Armachiho District, Northwest Ethiopia: a cross-sectional study. J Parasitol Res. 2018;2018:5751743. https://doi.org/10.1155/2018/5751743.**

**Ostyn B, Gidwani K, Khanal B, Picado A, Chappuis F, Singh SP, et al. Incidence of symptomatic and asymptomatic Leishmania donovani infections in high-endemic foci in India and Nepal: a prospective study. PLoS Negl Trop Dis. 2011;5: e1284.**

**https://doi.org/10.1371/journal.pntd. 0001284.**

In endemic areas, detection of antibodies might not be a surprise and signifies individuals’ exposure to the Leishmania donovani parasite. Those seropositive individuals can be (i) asymptomatic carriers and had spontaneously cured the parasite, (ii) asymptomatic individuals recently infected and not yet developed the disease, or (iii) individuals who had developed the disease and got cured following anti-leishmanial treatment (according to Porlino et al, 55% of treated VL patients were found seropositive). The latter is the case in which one of the participants in the present study had a VL history. Thus, detection of Leishmania may not be an appropriate phrase. This is further corroborated by studies that revealed the persistence of antibodies against rK39 in asymptomatic infections or previously infected patients for many months or years after the parasites have been cleared. These finds warrant the inaccuracy of using these tests to detect active infection. Further, the authors of this manuscript raised their concern in utilizing serodiagnosis given seropositivity may represent false positive cases due to cross-reactivity of antibodies with other pathogens in their discussion.

**Reply:**

**In line with your comment above, we have modified the overall recommendations of the paper.**

**We agree with you that the current definition of asymptomatic *Leishmania* infection is not ideal. This is in line with part of the discussion**

**“A basis limitation of all studies on asymptomatic Leishmania infection in general relates to the lack of a gold standard, and most studies have defined asymptomatic Leishmania infection as positivity on any of the Leishmania tests used [1]. The presence of Leishmania antibodies in the blood are an indirect marker for asymptomatic Leishmania infection and could merely be an indication of a past infection, which is now fully controlled or cleared, or could be due to cross-reactivity. Detection of parasite DNA in the blood at least indicates ongoing infection, as it has been shown that the DNA detected by PCR in the blood stems from viable parasites, and is degraded rapidly in the blood [2].**

**It is thus currently unclear at the global level which tests should be used to rule out asymptomatic Leishmania infection in blood donors.”**

**We agree that detection of antibodies against *Leishmania* does not mean there actually is a true Leishmania infection in the individual at the time of blood collection, as this could merely indicate previous exposure to the parasite, with clearance of the parasite later on.**

**Nevertheless, almost all studies on asymptomatic *Leishmania* infection use a similar definition, defining asymptomatic *Leishmania* infection as positivity on any of the *Leishmania* tests used, often combining direct and indirect tests.**

**Two recent review papers and one viewpoint paper have reflected on this a bit further**

**<https://pubmed.ncbi.nlm.nih.gov/34983616/>**

**“Common usage guidelines**

**Standardization of the term “asymptomatic Leishmania infection” is key to improving the outcome of future studies and allowing accurate comparison between the results of different works. According to the literature, subjects are often considered as asymptomatically infected with Leishmania under the following conditions: residence (or extended stay) in a Leishmania-endemic area, no reported signs/symptoms compatible with leishmaniasis, and positive on a combination of serological, molecular, cellular, and/or parasitological tests. We consider this an appropriate definition. While a medical history of leishmaniasis may complicate interpretation of certain tests, we feel it does not preclude the development of an asymptomatic infection if the subject was previously considered “cured.” More research regarding how many years following a reported VL episode a patient can be considered “asymptomatic” is necessary; as such, it is recommended that the conditions of a study population with VL history be explicitly stated. Furthermore, we recommend caution when comparing results of different studies on the subject of asymptomatic infections, as the reported prevalence cannot confidently be compared between areas due to the wide variety of tests employed by research groups. With respect to future studies, we suggest that researchers use the most sensitive and specific test available to them, and interpret their results within the framework of other studies employing the same technique and target. We recommend a minimum of two different detection methods employed in parallel for identification of asymptomatic Leishmania infections among a study population.**

**The definition of asymptomatic Leishmania infection is not unified across the literature, but often includes the following criteria: residence (or extended stay) in a Leishmania-endemic area, no reported signs/symp‑ toms compatible with leishmaniasis, and positive on a combination of serological, molecular, cellular, and/or parasi‑ tological tests.”**

**<https://pubmed.ncbi.nlm.nih.gov/32189034/>**

**“In summary, more research is needed to reach a consensus on how to define ‘asymptomatic’ and on the tools to identify carriers as the role they play in transmission may be of paramount importance for elimination/control programmes.”**

**“Since the results of using a single test to determine the prevalence of asymptomatic subjects are highly variable and imprecise [55], and in the absence of a gold standard, the combination of several serological/cellular/molecular approaches to accurately estimate the real prevalence of asymptomatic infection is recommended.”**

**<https://pubmed.ncbi.nlm.nih.gov/24585564/>**

**“Another problem is the absence of validated markers for asymptomatic L. donovani infection, as so far diagnostic assays for VL have been evaluated primarily on their capacity to detect clinical disease.”**

**“Therefore, scaling up efforts to improve the characterization of asymptomatic infection in endemic regions and establishing a standard case definition for leishmanial infection should be a priority.”**

Authors affiliations:

The affiliations of some authors are not correct. For example, Johan van Griensven’s affiliation is not the Department of Internal Medicine, University of Gondar, Gondar, Ethiopia.

**Reply: apologies for this error, we have corrected it.**

Abstract:

Lines 44-46. Please rephrase those sentences. As I pointed out above, detection of Leishmania is not appropriate it implies the detection of the Leishmania parasite or its component (e.g., amastigote, Leishmania specific antigen, or DNA). Detection of antibodies is a piece of indirect evidence regarding recent or past infections and may not necessarily indicate acute infection as described above. The authors’ recommendations also need to tone down as they are very challenging to implement in Ethiopian settings. Studies in Ethiopia, including areas where the present study was conducted, show that the majority (over 80%) of blood donors are males. In line with this, even systematic screening of male donors is logistically challenging.

**Reply: in line with your suggestion above, we agree implementation of this recommendation could lead to more harm than benefit and have modified the paper at various points in line with that.**

**We agree the current definition of asymptomatic *Leishmania* infection is cumbersome; given the lack of a better definition, there is no easy solution to that. We have modified the phrasing in lines 44-46.**

Summary:

Lines 58-61. I recommend those detailed results should be moved to the abstract rather than appearing in the summary.

**Reply: we have removed part of the details and shortened other parts, to reduce the overlap with the abstract.**

Line 61. “There were six patients …………………. Authors should avoid calling seropositive or DNA-positive individuals as patients. According to Transfusion Guidelines, blood donors are required to be apparently healthy. Therefore, those six positive donors in the present study should be considered as hypothetical asymptomatic VL cases.

**Reply: we agree. We have replaced “patients” by “individuals”**

<b>Summary and General Comments</b></br></br> Use this section to provide overall comments, discuss strengths/weaknesses of the study, novelty, significance, general execution and scholarship. You may also include additional comments for the author, including concerns about dual publication, research ethics, or publication ethics. If requesting major revision, please articulate the new experiments that are needed.

Reviewer #1: This study fills an important knowledge gap on prevalence of asymptomatic infection among blood donors in and near VL endemic areas in Ethiopia. Although findings are straight forward, the interpretation, and especially the possible implications for recommendations regarding routine screening of donor blood, remains difficult, and is appropriately discussed.

Comments:

- The reference numbers in the text do not correspond with the correct references in the References section.

**Reply: apologies for this error, something seems to have gone terribly wrong. We have corrected this.**

- Line 36 and 179: Asymptomatic infection is defined a positivity of any test. I suggest to add "in a health person".

**Reply: modified as suggested.**

- Line 72: the estimated annual VL incidence range of 3700 to 7400 is based on outdated estimations for underreporting from the early 00's of 2-4 times. WHO has published more recent estimations for underreporting of VL incidence in Ethiopia of 1.2 - 1.8 (WHO Weekly Epidemiological Record, No 22, 2016, 91, 287-296).

**Reply: thank you, we now refer to this WHO Record and two other papers on Ethiopia.**

**<https://parasitesandvectors.biomedcentral.com/articles/10.1186/s13071-015-0987-y>**

**https://apps.who.int/iris/bitstream/handle/10665/254451/WER9122.pdf?sequence=1&isAllowed=y**

**https://www.ncbi.nlm.nih.gov/pmc/articles/PMC4154678/**

- Line 272: Authors suggest the possibility of screening of donor blood with rK39 RDT in the resource-limited East African setting. This seems a bit random and opportunistic, considering that the rK39 RDT only identified a small proportion of all serological positive cases, and the fact that no parasite DNA was found in any of the rK39 RDT positive cases. What does a positive (or negative) rK39 RDT then tell us?

**Reply: Thank you for raising this important and valid point. In line with a comment from Reviewer 3, and after further reflections and discussions within the team and with other Ethiopian colleagues, we think this recommendation might do more harm than good and have rephrased the paper in line with that.**

- The overlap of positivity for the different tests is very low (no overlap between DAT and rK39, and only one between rK39 ELISA and PCR). This discrepancy between test results raises questions about the performance of the different tests to assess asymptomatic infection in healthy persons. The serological tests have been validated for diagnosis of infection in suspect symptomatic cases, who generally have high antibody titers, but not for confirming infection in asymptomatic persons with low antibody titers. I would like to see a discussion on the interpretation of the discrepancy of positivity results between the different tests.

**Reply: very valid point. This is basically a limitation of all studies on asymptomatic Leishmania infection, and has been emphasized recently in a systematic review [3]. We currently use different tests that have been designed to detect active disease (VL), and in the vast majority of studies on asymptomatic *Leishmania* infection, a combination of these tests is used to define asymptomatic *Leishmania* infection, while we currently lack a “gold standard” for this condition. While we recognize this limitation for others and all the other studies, we have not come across a valid improved definition. In most studies, discrepancy of the various tests was documented.**

**While we think this issue would merit a dedicated viewpoint paper, we have added some possible explanations in the discussion. As recommended by another reviewer, we have also added a Table describing the correlation between the different tests (Kappa statistic).**

Reviewer #2: Abstract

Page 3, line 36: correct to ' as positive by any of...'

**Reply: modified as suggested.**

Introduction:

Page 5, line 70: Correct ' In East Africa...' linguistically.

**Reply: this sentence has already been rephrased based on another comment.**

71: are these the total number of cases or incidence rate per 100,000. Correct!

**Reply: this is the total number of cases. We have rephrased that sentence to make that clear.**

Page 5, line 76: What is meant by 'others'? Is it immunocompromised? Mention it/

**Reply: we have rephrased this to clarify that most individuals will remain asymptomatic, but the risk of progression to disease (VL) is higher in HIV infected individuals.**

Methodology

Page 7, line 106: better to mention the reference number of IRB approval

**We have added this information.**

Page 7, line 123: you may use 'deferred' a common blood bank word used in such situations.

**Reply: Thank you for this suggestion, we have modified that.**

Page 8, line 134: Mention the name of the formula used in sample size calculation or cite a reference.

**Reply: this has been added.**

Page 8, line 139: what is a pretested questionnaire?

**Reply: Basically, we mean that a draft version was pretested using mock study participants with further improvements done as required.**

Page 8, line 141: was the whole blood EDTA? What is meant by the same needle? Do you mean the same phlebotomy/withdraw then distributed to different tubes?

**Reply: EDTA indeed; while the phlebotomy needle was in place, we took the blood directly for the different tubes**

Page 9, line 150: what is the rationale behind using three serological tests?

**Reply: most studies have used different Leishmania tests to detect asymptomatic Leishmania infection, and in a recent review [3], this was confirmed as recommendation. This is probably due to the fact that in most studies, there has been a rather poor correlation between the different serological and other tests. Hence the idea that combining different tests would increase the sensitivity. However, as we mention above, we (and many authors) feel there are serious concerns with the current definition/approach but given a “gold standard” to define asymptomatic infection – even more as this concept currently covers a wide variety of “states” such as individuals that were infected but cleared the parasite, those with latent infection, and more – it remains unclear what is the best way forward. So in conclusion, our approach is in line with the vast majority of other studies on asymptomatic Leishmania infection, but we (and many others) acknowledge this is far from ideal but it is current practice and it what was recently advised in a systematic review on this matter.**

Page 9, line 161: correct to 'optical density, OD'

**Reply: modified as suggested.**

162" correct to ' 20% variability between the two reading..' or similar

**Reply: modified as suggested.**

Page 9, line 165: Did you use 300ul of whole blood or buffy coat, where we expect amastigotes to be?

**Reply: we used whole blood .**

Results

Table 1: re-edit the table according to journal style.

There is no need to include the P-value as it had no meaning here. The 2x2 table should give difference between VL and non-VL versus the exposure or variable like sex, male female.

**Reply: we have removed the P-values. We have tried to adapt the table. While double checking the author guidelines, we think the tables are in line with these but please let us know if further modifications would be needed.**

Page 12, Line 208: in a real laboratory protocol, one test should be used, which of the four is recommended?

**Reply: Please see our reply to one of your comments above (reply also pasted below here)**

**“Most studies have used different Leishmania tests to detect asymptomatic Leishmania infection, and in a recent systematic review, this was confirmed. This is probably due to the fact that in most studies, there has been a rather poor correlation between the different serological and other tests. Hence the idea that combining different tests would increase the sensitivity. However, as we mention above, we (and many authors) feel there are serious concerns with the current definition/approach but given a “gold standard” to define asymptomatic infection – even more as this concept currently covers a wide variety of “states” such as individuals that were infected but cleared the parasite, those with latent infection, and more – it remains unclear what is the best way forward. So in conclusion, our approach is in line with the vast majority of other studies on asymptomatic Leishmania infection, but we (and many others) acknowledge this is far from ideal but it is current practice and it what was recently advised in a review on this matter.”**

**Two recent review papers and one viewpoint paper have reflected on this a bit further**

**<https://pubmed.ncbi.nlm.nih.gov/34983616/>**

**“Common usage guidelines**

**Standardization of the term “asymptomatic Leishmania infection” is key to improving the outcome of future studies and allowing accurate comparison between the results of different works. According to the literature, subjects are often considered as asymptomatically infected with Leishmania under the following conditions: residence (or extended stay) in a Leishmania-endemic area, no reported signs/symptoms compatible with leishmaniasis, and positive on a combination of serological, molecular, cellular, and/or parasitological tests. We consider this an appropriate definition. While a medical history of leishmaniasis may complicate interpretation of certain tests, we feel it does not preclude the development of an asymptomatic infection if the subject was previously considered “cured.” More research regarding how many years following a reported VL episode a patient can be considered “asymptomatic” is necessary; as such, it is recommended that the conditions of a study population with VL history be explicitly stated. Furthermore, we recommend caution when comparing results of different studies on the subject of asymptomatic infections, as the reported prevalence cannot confidently be compared between areas due to the wide variety of tests employed by research groups. With respect to future studies, we suggest that researchers use the most sensitive and specific test available to them, and interpret their results within the framework of other studies employing the same technique and target. We recommend a minimum of two different detection methods employed in parallel for identification of asymptomatic Leishmania infections among a study population.**

**The definition of asymptomatic Leishmania infection is not unified across the literature, but often includes the following criteria: residence (or extended stay) in a Leishmania-endemic area, no reported signs/symp‑ toms compatible with leishmaniasis, and positive on a combination of serological, molecular, cellular, and/or parasi‑ tological tests.”**

**<https://pubmed.ncbi.nlm.nih.gov/32189034/>**

**“In summary, more research is needed to reach a consensus on how to define ‘asymptomatic’ and on the tools to identify carriers as the role they play in transmission may be of paramount importance for elimination/control programmes.”**

**“Since the results of using a single test to determine the prevalence of asymptomatic subjects are highly variable and imprecise [55], and in the absence of a gold standard, the combination of several serological/cellular/molecular approaches to accurately estimate the real prevalence of asymptomatic infection is recommended.”**

**<https://pubmed.ncbi.nlm.nih.gov/24585564/>**

**“Another problem is the absence of validated markers for asymptomatic L. donovani infection, as so far diagnostic assays for VL have been evaluated primarily on their capacity to detect clinical disease.”**

**“Therefore, scaling up efforts to improve the characterization of asymptomatic infection in endemic regions and establishing a standard case definition for leishmanial infection should be a priority.”**

Page 13, line 214: what was the new stringent definition used?

**Reply: this used more strict cut-offs to define a positive case. We included this information in the methods section.**

Table 2: edit table.

Is such studies the difference is established between diseased (VL) and non-diseased (non-VL) with suspected exposure/risk factor? It will not be useful to compare between the two hospitals.

Cross-sectional study are problematic for low prevalence diseases as the number of positive cases will be low for statistical inference, which is 23/426 in this case.

**Reply: we have edited the table. Comparison between those with or without asymptomatic infection is done in Table 3. Is your suggestion to add a column in that table (one column “asymptomatic Leishmania infection”, the other “no asymptomatic Leishmania infection”? Or for each marker, to look at the association with exposure/risk factors?**

**The purpose of Table two is to compare the markers between a hospital in a VL endemic area and a hospital next to a VL endemic are. For instance, in the former, one would expect more PCR positivity but given the increasing expansion of VL to previously non-endemic areas, we thought it would still be useful to look at this.**

Page 14, line 227: Metema is VL-endemic area. Gondar is a VL non-endemic area. In light of this, the results are not surprising and expected. Look at the data differently as in mentioned in my comments above.

**Reply: apologies, but we were not entirely clear how to modify the analysis beyond the changes done in this revised version. We hope the modifications we did in the paper are sufficiently addressing your comments?**

Table 3: see comments on table 2.

**While double checking the author guidelines, we think the tables are in line with these but please let us know if further modifications would be needed.**

Discussion

Page 17, line 245: High among males above 35. Might be that males are the main donors compared females. This the age of donation in that area. This could be the explanation. Check!

**Reply: Males indeed constituted the majority of individuals included in the study (59%). Other studies from the study area have already found males to be more likely to donate blood.**

**<https://www.dovepress.com/practice-of-blood-donation-and-associated-factors-among-adults-of-gond-peer-reviewed-fulltext-article-JBM>**

**But having more males in the study would not automatically imply that the prevalence of asymptomatic Leishmania infection would be higher in males. If there would be no association between these two, the prevalence would be the same in males and females. But males included in our study were found to have a higher prevalence of asymptomatic Leishmania infection. In addition, the highest prevalence was seen in the 35-45 age group. Combined, this contributes to the highest prevalence of asymptomatic Leishmania infection in males above 35 years old.**

**We hope this answers your concern. Our apologies if we did not understand your question correctly.**

Page 17. Line 259: Correct 'basis' to 'basic'.

**Reply: modified as suggested.**

Page 18, line 272: In this study rk39 ELISA detect the highest number of symptomatic VL cases. Thus should be adopted! or perhaps IFAT or ELISA as they are adopted for the WHO VL case definition.

**Reply: we have now stressed the highest detection rate of rK39 ELISA in the discussion.**

Page 19, line 285: 'blood is scarce' do you mean blood donation?

**Reply: this has been clarified; indeed supplies of donated blood are still limited**

Page 19, line 294: Correct 'DND' to 'DNA'.

**Reply: modified as suggested.**

Page 19, 295: rephrase the sentence 'On the other hand....'.

**Reply: we were not entirely clear how you would like to see the sentence rephrased. As the sentence was quite long, we turned it into two sentences. If additional changes are needed, please let us know.**

Page 20, 309: another limitation is the low number of VL (23/426) to draw statistical conclusion. This is a general problem in cross-sectional studies targeting low prevalence disease.

**Reply: correct, we have added this as a limitation.**

Figures

Fig 1: Good representation. Agreement can also be tested by kappa statistic which will make the figure stronger.

**Reply: we have added a new table displaying the kappa statistic**

Reviewer #3: Given the high prevalence of immunocompromised individuals as the result of HIV/AIDS infection in Ethiopia and the limited or no data availability on the prevalence of asymptomatic L. donovani infection in Ethiopia, studies using highly sensitive and specific molecular assays over serological-based studies are of importance to evaluate the risk of Leishmania parasites infection in the blood donors. The present study by Mohammed et al study aimed to provide additional data on asymptomatic Leishmania donovani infection among Ethiopian blood donors residing in VL endemic and proxy regions to provide additional evidence for potential transmission L. donovani to blood transfusion recipients, particularly immunosuppressed individuals. However, like that of several similar studies that have been done in many VL endemic countries, this study had not proven the transmission of L. donovani by blood transfusion. To prove the possibility of L. donovani transmission via a blood transfusion to susceptible recipients, the authors could have employed a cohort study design that includes the follow-up of both blood donors and recipients for several years for the development of laboratory-confirmed clinical signs and symptoms compatible with VL using highly sensitive and specific molecular assays which are reliable for making decision active infection accurately. Additionally, a viability and infectivity test of L. donovani parasites in frozen blood products in blood banking procedures and storage conditions is required.

**Reply: thank you for these very important points. We agree more research is needed to better define the risk of Leishmania transmission and have integrated your suggestions in the discussion.**

--------------------

PLOS authors have the option to publish the peer review history of their article (<a href="[https://journals.plos.org/plosntds/s/editorial-and-peer-review-process#loc-peer-review-history](https://journals.plos.org/plosntds/s/editorial-and-peer-review-process#loc-peer-review-history)" target="_blank">what does this mean?</a>). If published, this will include your full peer review and any attached files.<br><br>

If you choose “no”, your identity will remain anonymous but your review may still be made public.<br><br>

<b>Do you want your identity to be public for this peer review?</b> For information about this choice, including consent withdrawal, please see our <a href="<https://www.plos.org/privacy-policy>" target="_blank">Privacy Policy</a>.

Reviewer #1: No

Reviewer #2: No

Reviewer #3: No

Figure Files:

While revising your submission, please upload your figure files to the Preflight Analysis and Conversion Engine (PACE) digital diagnostic tool, <https://pacev2.apexcovantage.com>. PACE helps ensure that figures meet PLOS requirements. To use PACE, you must first register as a user. Then, login and navigate to the UPLOAD tab, where you will find detailed instructions on how to use the tool. If you encounter any issues or have any questions when using PACE, please email us at [figures@plos.org](mailto:figures@plos.org).

Data Requirements:

Please note that, as a condition of publication, PLOS' data policy requires that you make available all data used to draw the conclusions outlined in your manuscript. Data must be deposited in an appropriate repository, included within the body of the manuscript, or uploaded as supporting information. This includes all numerical values that were used to generate graphs, histograms etc.. For an example see here: <http://www.plosbiology.org/article/info%3Adoi%2F10.1371%2Fjournal.pbio.1001908#s5>.

Reproducibility:

To enhance the reproducibility of your results, we recommend that you deposit your laboratory protocols in protocols.io, where a protocol can be assigned its own identifier (DOI) such that it can be cited independently in the future. Additionally, PLOS ONE offers an option to publish peer-reviewed clinical study protocols. Read more information on sharing protocols at <https://plos.org/protocols?utm_medium=editorial-email&utm_source=authorletters&utm_campaign=protocols>

__________________________________________________

In compliance with data protection regulations, you may request that we remove your personal registration details at any time. (Use the following URL: <https://www.editorialmanager.com/pntd/login.asp?a=r>). Please contact the publication o

1.	Michel G, Pomares C, Ferrua B, Marty P. Importance of worldwide asymptomatic carriers of Leishmania infantum (L. chagasi) in human. Acta Trop. 2011;119(2-3):69-75.

2.	Prina E, Roux E, Mattei D, Milon G. Leishmania DNA is rapidly degraded following parasite death: an analysis by microscopy and real-time PCR. Microbes Infect. 2007;9(11):1307-15.

3.	Ibarra-Meneses AV, Corbeil A, Wagner V, Onwuchekwa C, Fernandez-Prada C. Identification of asymptomatic Leishmania infections: a scoping review. Parasit Vectors. 2022;15(1):5.
