# Supplementary material for: Detection of asymptomatic Leishmania infection in blood donors at two blood banks in Ethiopia
Source: PLoS Negl Trop Dis. 2023 Mar 9;17(3):e0011142. doi: 10.1371/journal.pntd.0011142 (PMC10030029; doi:10.1371/journal.pntd.0011142)
Supplement: Attachment — Submitted filename: Blood_Tx_R2_Reply.docx [file pntd.0011142.s003.docx]

**Point by point response to the reviewers’ comments**

**Dear reviewers,**

**Thank you again for your careful review of our manuscript. We hope we have addressed all points in a satisfactorily way. When referring to line numbers below, please not that this refers to the version with track-changes.**

Reviewer #2: The Authors have corrected all points of concern, however, Odds ratio should be included in the 'statistics' section.

**Reply: We have added this in that section (line 206-207).**

-	Line 143: It is not clear why the rK39 RDT was not done at Metema immediately after sample collection as was done in Gondar.

**Reply: we preferred to have all tests done in Gondar, as having the same test done at two different sites could add additional variability. We have documented before that test performance is not affected by short storage of samples before testing.**

-	The definitions for the main analysis and secondary analysis are not clear in the methods and the results sections. Perhaps, the manuscript will be clearer if these outcome analyses are defined and described in a separate subtitle. Are these definitions applicable only for DAT and PCR, or used in composite analysis with other test results too? The results section also does not clearly show the findings of main analysis and secondary analysis. For example: there are 2 DAT positive results (Table 2). Are these based on the main analysis (1:1600) or secondary analysis (1:3200)?

**We now see that the way it was presented could indeed be confusing. Basically, what we did is that we defined asymptomatic infection as mentioned in the methods and then conducted all analysis using this definition. So all data shown relate to this. Finally, we additionally calculated prevalence using a more stringent definition. This is mentioned in one line under the ‘Prevalence of asymptomatic infection’ heading in the Results.**

**So introducing the term “secondary analysis” for this alternative way to calculate prevalence is perhaps a bit of overshooting and confusing.**

**We have now moved all the explanation to the statistics section, without referring to “secondary analysis” but providing the information above (lines 201-205).**

-	The CT cut off value used to define the PCR result as positive or negative is not clear. It is stated that the analysis was repeated when the CT values were >35. On the other hand, the median CT value for the PCR positives was 40.4 (39.6 – 40.6) indicating majority had CT values >35.

**Reply:**

**No cut-off was used for the PCR, rather samples were repeated when a Ct value was above 35 and only called positive when the repeat also gave a signal. Indeed most positive samples in our study have been repeated, as Ct values were generally high.**

**For all samples with Ct values above 35, a repeated extraction and PCR was done to confirm positivity. If the repeat test was positive again, the Ct of the first run was used. In case this result was not confirmed in the second run, the sample was called weak positive and Ct of the first run was used for the analyses.**

**We have rephrased this in the methods to make it more clear (lines 187-190.**

Reviewer #2: All were corrected, however Tables 2 and 3: What is the statistical tool used to calculate the P-value. Mention it below the table.

In both tables correct to ‘P-value’

**We have added this information under the tables and in the methods section**

Reviewer #4: -	Line 259-60: it is mentioned that lower levels of antibodies can be expected in asymptomatic infections. On the flip side, asymptomatic infection may actually have higher antibodies due to their strong immunity protecting them from developing the disease. In fact, the discrepancy between the DNA detection and antibody detection may indicate that those with positive test for antibody detection have cleared parasites than those who tested positive with PCR. These arguments need to discussed in parallel and supported by references if available.

**To the best of our knowledge, available data show lower levels of antibodies in asymptomatic infection compared to VL cases.**

**For instance:**

[**https://pubmed.ncbi.nlm.nih.gov/31649631/**](https://pubmed.ncbi.nlm.nih.gov/31649631/)

**Also in Ethiopia (PreLeish), optic densities for rK39 ELISA were around six times higher for VL cases compared to asymptomatic individuals in our recently finalized PreLeish study** [**https://clinicaltrials.gov/ct2/show/NCT03013673**](https://clinicaltrials.gov/ct2/show/NCT03013673) **(manuscript in preparation).**

**Protective immunity against VL is as far as we know mainly based on the cellular immune response (Th1) and not (or hardly) based on the humoral response (Th2). In that sense, higher antibody levels could be an indication of a weaker Th1 response and a stronger Th2 response.**

**In several studies in the Indian subcontinent, higher levels of anti-leishmania antibodies (DAT & rK39 ELISA) have been found to be associated with an increased risk of VL progression.**

**For instance:** [**https://pubmed.ncbi.nlm.nih.gov/24466361/**](https://pubmed.ncbi.nlm.nih.gov/24466361/)

**Conversely, a positive cellular test (whole blood assay), as an indicator of the cellular/Th1 response was found to indicate a very low risk of progression to VL in other studies.**

[**https://pubmed.ncbi.nlm.nih.gov/31164191/**](https://pubmed.ncbi.nlm.nih.gov/31164191/)

[**https://www.ncbi.nlm.nih.gov/pmc/articles/PMC6453476/**](https://www.ncbi.nlm.nih.gov/pmc/articles/PMC6453476/)

**As we think there is not much evidence supporting the notion of higher levels of *Leishmania* antibodies in asymptomatic infection and that the presence of anti-leishmania antibodies – as an indicator of stronger immunity – could explain/lead to clearance of parasite DNA, we would prefer not to expand on this in the discussion.**

Reviewer #4: Abstract

-	Line 42: It does not make sense to analyze for association between history of VL with asymptomatic infection as there was only one person with history of VL in the data.

**We have rephrased this in the abstract and the results.**

Introduction

-	Line 86: insert reference for the sentence, “Transmission of VL via blood transfusion has been demonstrated in hamsters and dogs”

**We have inserted two references.**

Reviewer #3:

Nevertheless, authors have adequately addressed most of my prior comments, suggestions and questions. However, I have a couple of comments which authors need consider.

1. In principle, the prevalence of asymptomatic and symptomatic L. donovani infections is higher endemic areas than in non-endemic areas. Given this fact, using the same prevalence rate for estimation of the sample sizes for two study sites (Metema and Gondar) doesn’t sound statistically and scientifically. I still think the sample size calculation is misleading and a better explanation for using the same sample size for two study sites should be provided. If not, this has to be stated, in the revised manuscript, as one of the limitations of the study.

**We have added this as a limitation (lines 347-351).**

2. Authors pointed out the expansion of VL endemicity form historically VL endemic areas to previously nonendemic highland areas (e.g., Libo Kemkem) in Ethiopia possibly due to global warming and considered this as their additional reason for enrolling number of blood donors from Gondar. On the contrary, throughout their manuscript they keep saying nonendemic area. There is a suspect for occurrence local L. donovani transmission in Godar city and its surroundings due to documented hypothetical autochthonous cases that never had a travel history to the known VL endemic areas although it has not yet properly Investigated. I therefore kindly recommended authors to replace "VL nonendemic area" with” previously VL non-endemic area”.

**Thank you, this is a fair point. Modified as suggested.**

3. The explanation that DAT has historically been developed for detection of symptomatic VL doesn’t sound. Because this explanation does also apply to rK39 ELISA as it also has originally developed and validated to detect symptomatic VL infections. In principle, DAT is meant to detect anti-leishmania antibodies. Similarly, rK39 ELISA also detects anti-leishmania antibodies. It is true that different levels of antibody response can be found in asymptomatic infections verses in symptomatic L. donovani infections- possibly higher level in VL patients and lower in individuals with asymptomatic L. donovani infection. The most plausible reason for relative higher sensitivity of rK39 ELISA as compared to DAT in detection of asymptomatic infection could be due to the high immunogenicity of the targeted antigen and the prolonged presentence of anti-rK39 antibodies. Discuss this.

**We agree that both rK39 ELISA and DAT have historically been developed for VL diagnosis. We have integrated your suggestion in the discussion (lines 285-287).**

Reviewer #4: This is a well written article in an area which is not well investigated. The researchers have come up with an important issue that needs attention. Such studies on transmission mechanisms of neglected infectious diseases will help in the overall control and elimination strategies. I believe that the authors have addressed previous reviewers' comments appropriately. I have a few comments see above under the different sections.

**Thank you for your comment.**
